# Gene Expression Signature of a Fibroblast Serum Response Predicts Cancer Progression

**DOI:** 10.1371/journal.pbio.0020039

**Published:** 2004-01-13

The idea that cancer cells go through a fateful transition that turns them into fast-growing, invasive, metastasizing tumors first surfaced in the early 1970s. During this conversion, blood vessels form around the tumor, providing a dedicated supply of blood to fuel the tumor's aggressive behavior. By the mid-1980s histological analysis revealed a similarity between the tumor “microenvironment” and that of a healing wound, prompting Harvard pathologist Harold Dvorak to describe cancer as a wound that does not heal. When the body sustains a wound, it coordinates an emergency response defined by rapid cell proliferation, invasion and “remodeling” of connective tissues and extracellular matrix (the network of proteins and molecules around cells), cell migration, and blood vessel formation (angiogenesis). These processes, which are restorative in normal wound healing, may promote cancer by supporting tumor formation, invasion, and metastasis. With no systematic method to measure the “wound-like” features in cancer, however, scientists have no way to evaluate the risk that a wound-healing genetic program may pose in cancer progression.

A molecular understanding of the wound-healing process and its connection to cancer would provide insight into the nature of these similarities and perhaps provide molecular indicators of tumor progression. In an effort to create a framework for evaluating this relationship, Howard Chang and his colleagues at Stanford University developed a model to predict cancer progression based on the gene expression profile of a cellular response to serum in cell culture.

Part of the problem with evaluating the physiological status of a tumor based on its genetic profile is that current techniques indicate only the expression, not the effect, of genes. To develop a strategy for interpreting biological outcomes from a gene expression profile, Brown's team modeled a physiological process by exposing cultured fibroblasts to serum—the soluble fraction of coagulated blood—and tracking gene expression. Serum is encountered in the body where blood leaks out of blood vessels (in essence, all the sites of injury) and is thought to be a major initiator of the wound response. Fibroblasts exist in the connective tissue of epithelial organs (which include the digestive tract, lungs, and mammary glands) and contribute to organ development, wound healing, inflammation, and a condition called fibrosis. (Fibrosis involves the same type of extracellular matrix remodeling seen in wound healing and cancer.) And fibroblasts can promote tumor formation and metastasis when mixed with epithelial cancer cells.

Though fibroblasts from different sites in the body differ in their properties and gene expression profiles, Chang et al. found that they share a common expression pattern in response to serum. From this expression profile, the researchers identified a core group of genes—a genetic signature—associated with a serum response. Because many of the genes in the signature were known to be involved in various wound-healing processes—such as matrix remodeling, cell motility, and angiogenesis—Chang et al. used this signature as a surrogate marker to measure how much tumors may be like wounds. When they compared the wound-like genetic signature with the expression profiles of various clinical tumor samples, they found the signature was always present in certain cancers—prostate and liver-cell carcinomas—and occurred variably in others—breast, lung, and gastric carcinomas. In each of these three latter types of tumors, patients with tumors carrying the serum-activated wound-like genetic signature had a significantly increased risk of metastasis and death compared to patients with tumors that lacked the signature. Therefore, Chang et al. conclude that a wound-like phenotype is a general risk factor for metastasis and the aggressive behavior in many of the most common cancers.

These results reveal a robust and useful similarity between the molecular programs in normal wound healing and tumor progression and metastasis. Although Chang et al. point out that their results do not indicate whether this fibroblast “fingerprint” is merely a marker for cancer progression or plays a role in orchestrating this pathway, they conclude that the genetic program activated in response to serum also contributes to tumor invasion and metastasis. This serum-response expression profile, the authors propose, provides a valuable new tool for predicting tumor behavior and determining a patient's prognosis.

**Figure:**
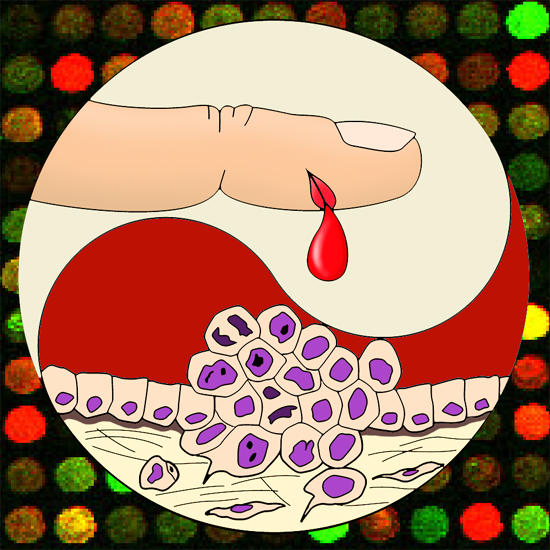
Genomics predicts tumor behavior

